# Hydrofluorocarbons pneumonitis as a complication of inhalation injury following air‐conditioning repairs

**DOI:** 10.1002/rcr2.983

**Published:** 2022-06-07

**Authors:** Afifah Aqilah Abdul Malik, Boon Hau Ng, Nik Nuratiqah Nik Abeed, Mohamed Faisal Abdul Hamid, Andrea Yu‐Lin Ban

**Affiliations:** ^1^ Pulmonology Unit, Department of Internal Medicine, Faculty of Medicine Universiti Kebangsaan Malaysia Medical Centre Kuala Lumpur Malaysia

**Keywords:** air conditioning, hydrofluorocarbons, inhalation injury, pneumonitis, systemic steroid

## Abstract

Hydrofluorocarbon (HFC) pneumonitis is an uncommon cause of inhalation injury. HFCs are a group of chemicals predominantly used for refrigeration and cooling. A 19‐year‐old air‐conditioning technician developed acute onset of dyspnoea and chest tightness while servicing an air conditioner in a confined space. We diagnosed him with HFC pneumonitis based on the history of exposure and the high‐resolution computed tomography (HRCT) thorax findings. He was treated with steroids and supportive oxygen therapy. He recovered fully after 5 days of hospitalization and was discharged. Review at 2 weeks in the outpatient setting showed significant radiological improvement on HRCT thorax.

## Graphical abstract

Hydrofluorocarbon (HFC) pneumonitis is an uncommon cause of inhalation injury. We report a unique case of acute HFC pneumonitis in a 19‐year‐old man following an inhalation injury during air‐conditioning service in a confined space.

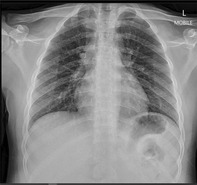

## INTRODUCTION

Inhalation injury refers to damage to the respiratory tract or lung tissue from heat, smoke or chemical irritants during inspiration that can cause acute and fatal pneumonitis.^1^ Hydrofluorocarbons (HFCs) are synthetic organic compounds that contain fluorine and hydrogen atoms.^2^ They are relatively non‐flammable and are used in a wide variety of cooling systems, from refrigerators and freezers to automotive air‐conditioning units. We report a unique case of acute HFC pneumonitis in a 19‐year‐old man following an inhalation injury during air‐conditioning service in a confined space.

## CASE REPORT

A previously well, non‐smoker, 19‐year‐old part‐time air‐conditioning service operator presented with an acute onset of dyspnoea after servicing an air conditioner in a confined closed office space. He noted a faint sweetish smell when he started his job but he continued to work. One hour later, he developed shortness of breath with palpitations and a sensation of chest tightness.

Upon arrival to the emergency department, pulse rate was 125/min with a respiratory rate of 22 breaths per minute (bpm) and oxygen saturation was 90% on room air. Examination of the respiratory system revealed generalized rhonchi with reduced air entry bilaterally. Systems review was unremarkable.

Laboratory results revealed elevated white blood cells (11.6/μl) and C‐reactive protein was 0.83 mg/L. Serum electrolytes, hepatic and renal function tests were normal. Arterial blood gas on room air demonstrated pH 7.39, partial pressure of carbon dioxide (PaCO_2_) 39.7 mmHg, partial pressure of oxygen (PaO_2_) 62.5 mmHg and HCO_3_ 23.6 mmol/dl. COVID‐19 real‐time reverse transcription‐polymerase chain reaction test was negative and electrocardiogram was normal.

**FIGURE 1:**
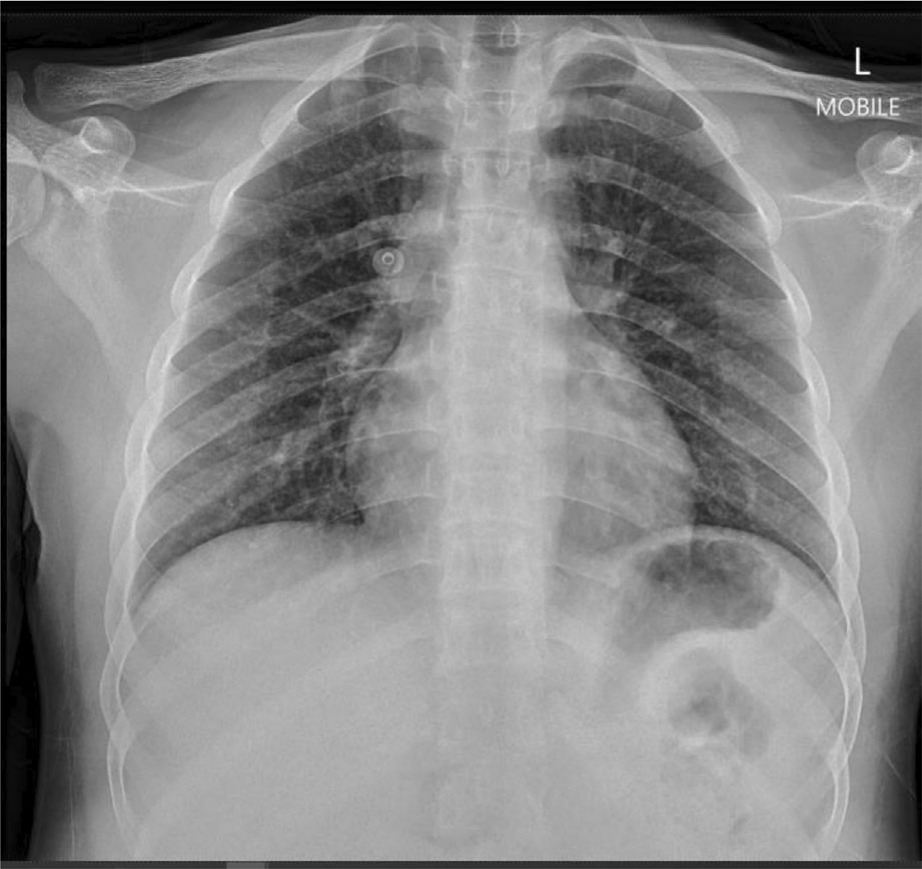
Chest radiograph on admission showing bilateral pulmonary infiltrates

**FIGURE 2:**
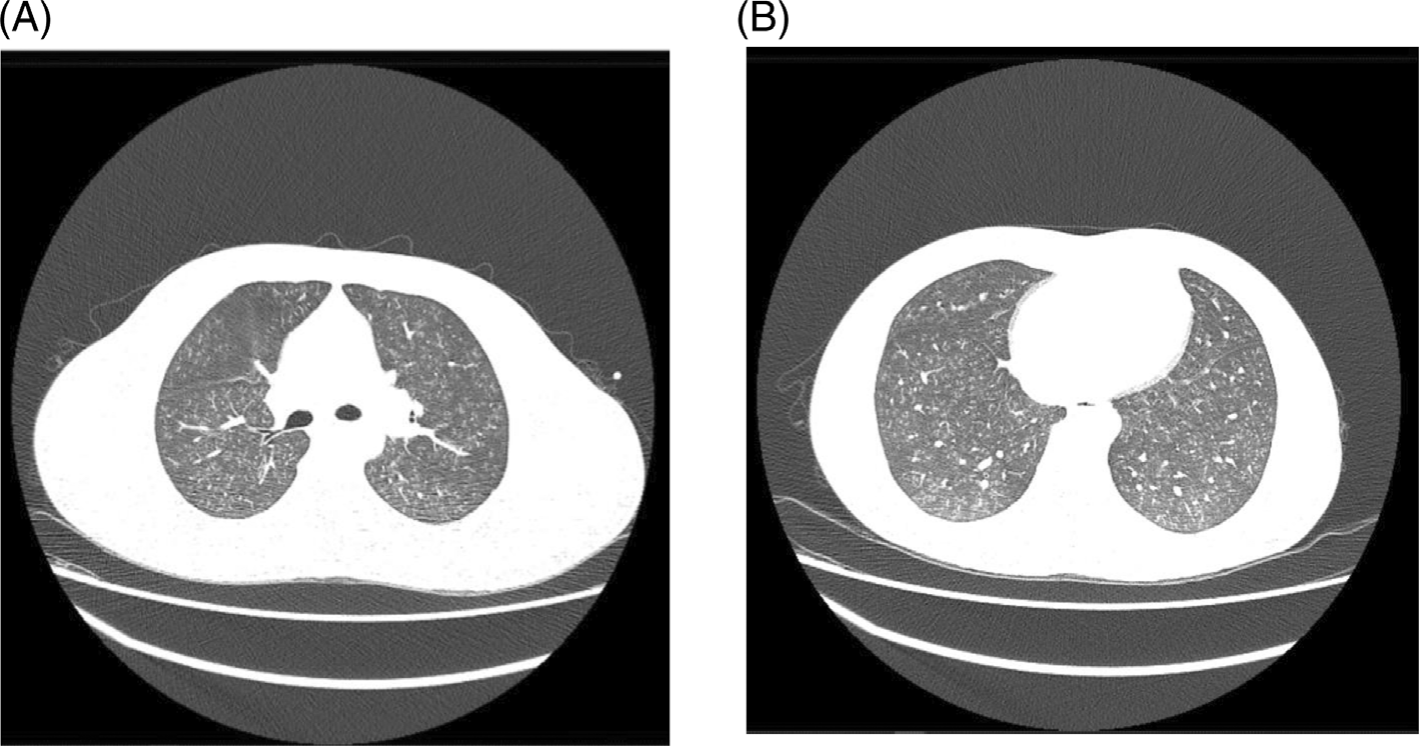
(A, B) High‐resolution computed tomography thorax (axial view) showed diffuse ground‐glass centrilobular nodules with minimal peripheral bronchiole wall thickening.

The chest radiograph showed mild pulmonary infiltrates (Figure 1). We proceeded with a high‐resolution computed tomography (HRCT) of the thorax that showed diffuse ill‐defined ground‐glass centrilobular densities scattered in both lung fields with no zonal predominance (Figure 2).

Based on the history and investigations, a diagnosis of hydrochlorofluorocarbon (HFC)‐induced acute pneumonitis was made. He was treated with 100 mg intravenous hydrocortisone three times per day, nebulized salbutamol and oxygen supplement. He was given intravenous antibiotics (amoxycillin and clavulanic acid) due to mild leucocytosis. Oxygen saturation improved markedly after the administration of intravenous steroid, and subsequently the patient was able to wean off oxygen at day 4 of intravenous steroid administration. He was discharged with oral prednisolone 35 mg daily for a total of 2 weeks. We did not perform spirometry or bronchoscopy.

**FIGURE 3:**
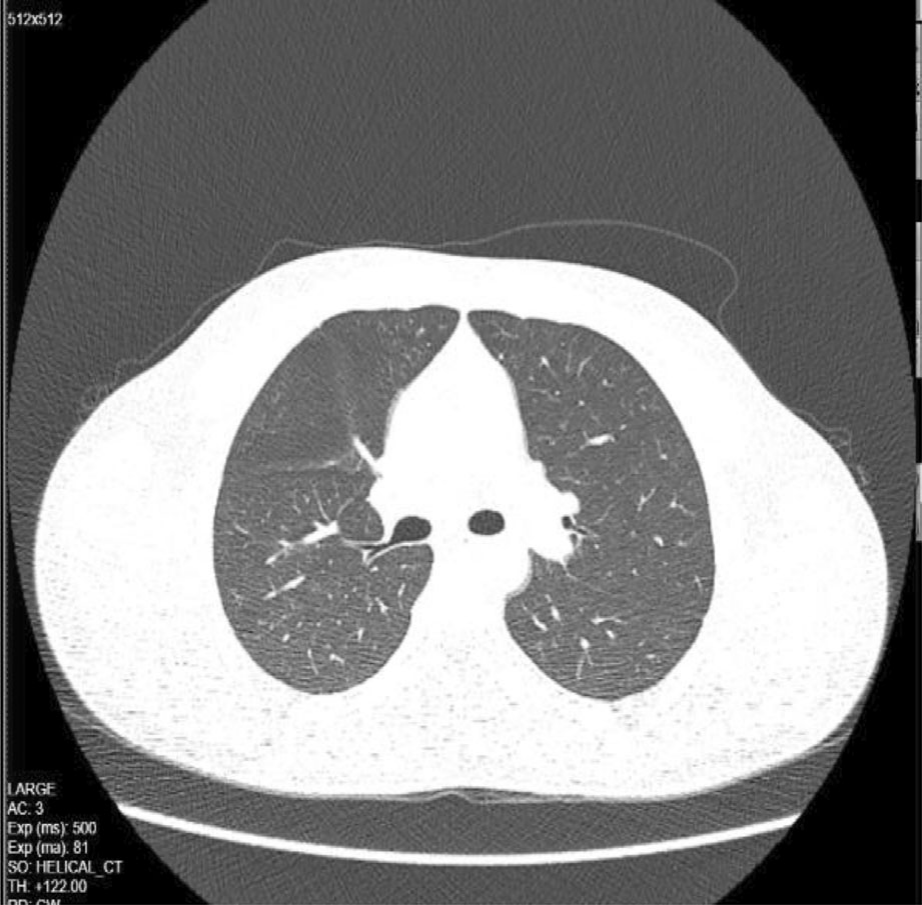
Marked improvement in centrilobular nodularity, with residual seen at the right upper and middle lobes

He was reviewed in clinic at 4 weeks post discharge. A repeat HRCT thorax (Figure 3) showed marked improvement in the degree of ground‐glass centrilobular nodules, with residual nodules seen at the right upper and middle lobes.

## DISCUSSION

HFCs are synthetic organic compounds that contain hydrogen, fluorine and carbons, which are relatively non‐flammable, chemically stable and non‐reactive.^2^ HFCs have a wide variety of usage and they are also frequently used in air conditioning and as refrigerants in refrigerators and freezers.^3^

Acute HFC exposure can cause multi‐organ dysfunction.^4^ HFC pneumonitis is an acute pneumonitis caused by aspiration and/or inhalation of HFC compounds with low viscosity and high volatility.^4^ The clinical presentation of hydrocarbon pneumonitis is often non‐specific and includes breathlessness, cough, chest pain and haemoptysis.^5^ This usually improves over a few days with supportive measures.^6^ The patient stated that he accidentally inhaled volatile HFCs during his work in an enclosed space and developed symptoms as described.

Exposure to several irritant substances at high levels may cause bronchiolitis and pulmonary oedema or ‘chemical pneumonitis’, depending on the solubility and physicochemical properties of the substance. HFCs disrupt surfactants, resulting in the decrease of pulmonary compliance and they can also cause pulmonary injury, with resultant inflammation, oedema and necrosis.^4^ The characteristic thin‐section computed tomography (CT) findings are bronchial wall thickening, centrilobular nodular areas of ground‐glass attenuation and confluent areas of ground‐glass attenuation with peribronchiolar distribution, or extensive ground‐glass opacity and consolidation.^7^ Some of these changes were evident in the case described.

A case report describing seven construction workers exposed following an installation of an additional HF (hydrofluoride) gas storage tank showed similar HRCT thorax findings of diffuse patchy ground‐glass opacities with centrilobular nodules. Treatment given was similar to our patient. They showed resolution of CT at 3 months.^8^ In this case, the follow‐up CT at 1 month showed remarkable improvement. However, no pulmonary function test and bronchoscopy were done for this patient.

Apart from supportive care, treatment with intravenous antibiotics and steroids are common therapies. Additionally, leucocytosis is commonly observed in patients with HFC pneumonitis, which is also a typical laboratory finding in cases of pneumonia.^9^ Differentiating chemical pneumonitis from infective pneumonitis is difficult but not impossible. Clinical history or further investigations, such as bronchoscopy, are useful. Sen et al. reported in their retrospective study that patients with hydrocarbon pneumonitis responded well to steroid therapy.^9^ However, none of these treatments have adequate randomized controlled data to prove their therapeutic effect on patients with hydrocarbon pneumonitis. Therefore, supportive care remains the mainstay of treatment for this condition.

In conclusion, HFC pneumonitis is an important diagnosis to make in a patient with a history of exposure to HFC products. Supportive care remains the mainstay of treatment, and antibiotics and steroids are reasonable therapeutic choices. Patients' clinical improvement precedes the resolution of radiological changes on chest x‐ray. The prognosis of patients with HFC pneumonitis appears favourable with accurate diagnosis and appropriate care.

## AUTHOR CONTRIBUTION

All authors contributed to the case report, discussion and writing of the manuscript.

## CONFLICT OF INTEREST

None declared.

## ETHICS STATEMENT

The authors declare that appropriate written informed consent was obtained for the publication of this manuscript and accompanying images.

## Data Availability

The data that support the findings of this study are available from the corresponding author upon reasonable request.

## References

[1] Woodson CL . Diagnosis and treatment of inhalation injury. In: Herndon DN , editor. Total burn care. 4th ed. Amsterdam: Elsevier; 2012.

[2] Rogers K . Hydrofluorocarbon. Encyclopedia Britannica. 2019 Aug 22. Available from: https://www.britannica.com/science/hydrofluorocarbon. Cited on Feb 2021.

[3] Shrivastava MS , Palkar AV , Karnik ND . Hydrocarbon pneumonitis masquerading as acute lung injury. BMJ Case Rep. 2011;2011:bcr0320114017. 10.1136/bcr.03.2011.4017 PMC3139168 22689551

[4] Tormoehlen LM , Tekulve KJ , Nañagas KA . Hydrocarbon toxicity: a review. Clin Toxicol. 2014;52(5):479–89. 10.3109/15563650.2014.923904 24911841

[5] Lee TH , Seymour WM . Pneumonitis caused by petrol siphoning. Lancet. 1979;314(8134):149. 10.1016/s0140-6736(79)90030-8 88581

[6] Akira M , Suganuma N . Acute and subacute chemical‐induced lung injuries: HRCT findings. Eur J Radiol. 2014;83(8):1461–9. 24853247 10.1016/j.ejrad.2014.04.024

[7] Lee Y , Jeong I . Chemical pneumonitis by prolonged hydrogen fluoride inhalation. Respir Med Case Rep. 2020;32:101338. 10.1016/j.rmcr.2020.101338 33489746 PMC7804834

[8] Hara M , Iwakami S , Sumiyoshi I , Yoshida T , Sasaki S , Takahashi K . Hydrocarbon pneumonitis caused by the inhalation of wood preservative. Respirol Case Rep. 2018;6:e00379. 10.1002/rcr2.379 30386622 PMC6203695

[9] Sen V , Kelekci S , Selimoglu Sen H , Yolbas I , Günes A , Abakay O , et al. An evaluation of cases of pneumonia that occurred secondary to hydrocarbon exposure in children. Eur Rev Med Pharmacol Sci. 2013;17(Suppl 1):9–12. 23436660

